# Nachwuchsförderung in der Anästhesiologie: attraktive Gestaltung der Famulatur

**DOI:** 10.1007/s00101-021-00936-5

**Published:** 2022-05-04

**Authors:** D. Scheffel, J. Wirkner, S. Adler, G. Wassilew, K. Dragowsky, R. Seemann, S. Fröhlich, AG-Lehre der DGOU, M. Ghanem, A. Meder, S. Bakir, B. Huoy, S. Herbstreit, R. Kasch

**Affiliations:** 1 Klinik und Poliklinik für Orthopädie und Orthopädische Chirurgie, Universitätsmedizin Greifswald, Ferdinand-Sauerbruch-Str., 17475 Greifswald, Deutschland; 2 Institut für Psychologie, Lehrstuhl für Physiologische und Klinische Psychologie/Psychotherapie, Universität Greifswald, Greifswald, Deutschland; 3 Klinik für Anästhesiologie, Anästhesie, Intensiv‑, Notfall- und Schmerzmedizin, Universitätsmedizin Greifswald, Greifswald, Deutschland; 4 Centrum für Muskuloskeletale Chirurgie, Charité – Universitätsmedizin Berlin, Augustenburger Platz 1, 13353 Berlin, Deutschland; 5 Orthopädische Klinik und Poliklinik, Poliklinik der Universitätsmedizin Rostock, Doberaner Str. 142, 18057 Rostock, Deutschland

**Keywords:** Medizinstudium, Lehre, Praktikum, Junge Anästhesisten, Schwerpunktwahl, Medical school, Teaching, Traineeship, Young doctors for anesthesiology, Medical specialist choice

## Abstract

**Hintergrund:**

Praktische Erfahrungen in Famulaturen können die spätere Weiterbildungswahl prägen.

**Fragestellung:**

Ziel der Untersuchung war es, Faktoren in der anästhesiologischen Famulatur zu finden, die Studierende ermutigen, sich auf das Fachgebiet zu spezialisieren.

**Material und Methoden:**

Im Rahmen einer bundesweiten Online-Umfrage beantworteten die Studienteilnehmenden (*n* = 479) Fragen zu ihrer mindestens 4‑wöchigen Famulatur in der Anästhesiologie. Die Befragten wurden in 4 Gruppen aufgeteilt: Diejenigen, die sich aufgrund der Famulatur ein Wahltertial im praktischen Jahr (PJ) in der Anästhesiologie vorstellen konnten (*n* = 212; 44 %), wurden mit denjenigen, die dies verneinten (*n* = 56; 12 %) und denjenigen, die sich schon vor der Famulatur festgelegt hatten (Ja: *n* = 144; 30 % und Nein: *n* = 67; 14 %) varianzanalytisch verglichen.

**Ergebnisse:**

Die Umfrage erreichte alle medizinischen Fakultäten in Deutschland und befragte Teilnehmende im durchschnittlichen Alter von 25,8 Jahren. In allen 4 ausgewerteten Gruppen fanden sich signifikante Unterschiede.

Die Studierenden, die mit der Famulatur zufrieden waren und sich für das PJ-Wahltertial in der Anästhesiologie aussprachen, unterschieden sich signifikant hinsichtlich Integration ins Team, Kompetenzerwerb, Struktur und Qualität der Lehre von den anderen Gruppen. Die Vermittlung von praktischen Kompetenzen und Fachwissen sowie die Integration in Diagnosefindung und Therapieplanung förderten ebenfalls die Nachwuchsgewinnung.

**Diskussion:**

Eine positiv bewertete anästhesiologische Famulatur fördert die spätere Spezialisierungspräferenz für dieses Fach. Für das Fachgebiet gewonnene Famuli erlangten mehr Fähigkeiten im Verlauf der Famulatur. Um angehende Ärzte für die Anästhesiologie zu gewinnen, sollte das ärztliche Team die oben genannten Kriterien bei der Famulaturgestaltung bedenken.

## Abstract (en)

**Background:**

Practical experiences in clinical traineeships can shape the later specialty choice of medical students.

**Key question:**

The following study aimed to find factors in anesthesiological clinical traineeship that encourage students to specialize in the field.

**Material and methods:**

As part of a nationwide online survey conducted by the working group for education of the German Association for Orthopedics and Trauma Surgery (Deutsche Gesellschaft für Orthopädie und Unfallchirurgie, DGOU), study participants (*n* = 479) answered questions about their minimum 4‑week traineeship in anesthesiology. The information on items was analyzed in six content categories: 1) integration into the team, 2) acquisition of skills, 3) teachers, 4) quality of teaching, 5) structure of teaching and 6) satisfaction with the clinical internship. The respondents were subdivided into 4 groups by answering the question “Could you imagine an elective in anesthesiology during the final year (PJ)” with “Yes, I have made this decision after the clinical traineeship” (JdF, *n* = 212, 44%), “No I have decided against an elective during the final year after the traineeship” (NdF, *n* = 56, 12%), “Yes I have decided for an elective in anesthesiology before the internship” (JvF Yes: *n* = 144, 30%) and “No, I have decided against an elective in anesthesiology before the internship” (NvF: *n* = 67, 14%). Answers of the participants regarding the six content categories were compared between the four groups.

**Results:**

The survey reached all medical faculties in Germany and included participants with an average age of 25.8 years and a balanced gender ratio. There were significant differences between satisfied and dissatisfied students in all four subgroups.

Of the 479 respondents, 211 (44%) were already set regarding their decision of choosing anesthesiology as an elective during the final year before the clinical traineeship. Of the respondents 268 (56%) were influenced by the internship, 212 (44%) of them positively. In total, 81% of the trainees rated the internship as “satisfying”. Students who were satisfied with the overall internship and who spoke in favor of the PJ elective in anesthesiology differed significantly from the other groups in the categories of team integration, skills acquisition, structure and quality of teaching.

The teaching of practical skills and specialist knowledge as well as the integration into diagnostics and treatment planning promoted the recruitment of young people.

**Discussion:**

The positively evaluated anesthesiology internship promotes later specialty choice, with quality and structure of the teaching affecting student satisfaction.

Trainees who were attracted by anesthesiology gave better overall ratings and acquired more skills during the course of the internship. In order to win aspiring doctors for anesthesiology, the medical team has to integrate trainees well and support the acquisition of practical skills and specialist knowledge. In addition, didactics and practical relevance should be given high priority.

## Hintergrund und Fragestellung

Nur wenige Studierende können sich schon zu Beginn des Studiums eine anästhesiologische Facharztweiterbildung vorstellen [1]. Will man sie im klinischen Abschnitt für die Anästhesiologie gewinnen, wird nach Mathis et al. (2016) wiederum nicht genügend Zeit eingeräumt, um dem umfangreichen Fach gerecht zu werden. Dabei könnten klinische Erfolgserlebnisse durch erlerntes und angewandtes Wissen besser für die Anästhesiologie begeistern als die theoretische Lehre [23]. Bekannt ist, dass Studierende bei der Wahl des Weiterbildungsfachs großen Wert auf praktische Erfahrungen legen und diese auch die Wahrnehmung des Fachs insgesamt positiv beeinflussen [3]. Somit kann im Rahmen des Zusammenspiels der curricularen Praktika insbesondere die Famulatur dazu dienen, Studierende frühzeitig für das Fach Anästhesiologie zu begeistern [3, 20]. Studien in Indien [21] und Australien [28] stellten bereits fest, dass praktisches Training das Interesse an Anästhesiologie als Facharztweiterbildung besonders stärkt. Außerdem wurde die positive Einstellung gegenüber dem Fach mit positiven Rollenmodellen assoziiert [21, 28]. Die Famulatur ermöglicht Studierenden, erste klinische Erfahrungen zu sammeln und Kontakte zu den klinisch tätigen Ärztinnen und Ärzten herzustellen [20].

In der Anästhesiologie herrscht, ebenso wie in vielen anderen Fachgebieten, ein Fachärztemangel. Dadurch bleiben viele Stellen unbesetzt [25]. Jede zweite anästhesiologische Abteilung kann, laut Forschungsgutachten der Deutschen Krankenhausgesellschaft [2], ihre offenen Stellen aufgrund von Bewerbermangel nicht besetzen. Ursachen für den wachsenden Ärztemangel seien ein erhöhter Anspruch an die Arbeitsbedingungen, „work-life balance“, Familienvereinbarkeit und die zunehmende Präferenz von Teilzeitarbeit sowie die Abwanderung in nichtkurative Arbeitsfelder und ins Ausland [14, 25]. Neben der Verhinderung von Abwanderungen in fachfremde (kurative/nichtkurative) Bereiche müsse die Rekrutierung angehender Ärztinnen und Ärzte als essenziell wahrgenommen werden [2, 25]. Die klinische, fachtheoretische Lehre in der Anästhesiologie wurde deutschlandweit bereits gut evaluiert und analysiert. So stellten Hoffmann et al. (2012) durch eine Befragung deutscher Lehrstühle fest, dass an vielen Fakultäten die anästhesiologische Lehre in Struktur, Inhalten und Umsetzungen übereinstimme und durch Synergien der Ordinariate sowie Fördergelder für die Wissenschaft gezielt verbessert werden könne [12]. Im Gegensatz zu anderen Fachgebieten, wie Orthopädie und Unfallchirurgie [20], Radiologie [19] und Chirurgie [5], mangelt es bezüglich curricularer Praktika in der Anästhesiologie bisher an Studien und einem nationalen Konsens über die Inhalte, die in der Famulatur vermittelt werden sollten.

Die Untersuchung stellte daher folgende Leitfrage: Inwiefern kann eine zufriedenstellende Famulatur Studierende für das Fachgebiet Anästhesiologie begeistern und frühzeitig ihr fachliches Interesse wecken? Die Sichtweisen der Studierenden wurden aus der bundesweiten Befragung Studierender durch die Arbeitsgemeinschaft Lehre (AG Lehre) der Deutschen Gesellschaft für Orthopädie und Unfallchirurgie (DGOU) abgeleitet.

Dabei wurde die Hypothese aufgestellt, dass die im Folgenden genannten Merkmale der Famulatur die spätere Wahl des PJ-Tertials in der Anästhesiologie beeinflussten. Zur Untersuchung dieser Hypothese wurden die Merkmale in 6 Themenbereiche eingeteilt: 1. die Integration der Famuli in das ärztliche Team, 2. der Erwerb von Kompetenzen im Verlauf der Famulatur, 3. die Lehrenden, 4. die Bewertung der Qualität, 5. die Bewertung der Struktur in der praktischen Lehre sowie 6. der Zusammenhang zwischen der Gesamtzufriedenheit im Praktikum mit den vorab getroffenen Aussagen. Die Ergebnisse der Studie sollen Anregungen liefern, ob und wie es gelingen kann, Studierende für das Fach Anästhesiologie zu begeistern und dadurch Nachwuchs in der Weiterbildung für Anästhesiologie zu gewinnen. Es sollen Ansatzpunkte für eine qualitative Verbesserung der Famulatur formuliert werden und der Vergleich zu Famulaturen in anderen Fachgebieten erfolgen.

## Studiendesign und Untersuchungsmethoden

Von April bis September 2012 nahmen an einer anonymisierten Online-Befragung der AG Lehre der DGOU bundesweit 9079 Medizinstudierende teil. Sie äußerten sich zu Erfahrungen während curricularer Praktika im Medizinstudium sowie ihren Erwartungen an den späteren Arbeitsplatz [16–18]. Die Studiendekanate oder Fachschaften aller medizinischen Fakultäten stellten initial den Kontakt zu den Teilnehmenden her.

EvaSys Education (Fa. Electric Paper Evaluationssysteme GmbH, Lüneburg, Deutschland), eine Software für Online-Befragungen, diente der statistischen Datenerfassung. Die finale Umfrage wurde durch die lokale Ethikkommission geprüft und befürwortet. Es wurden sowohl Studierende aus der Vorklinik, dem klinischen Abschnitt und Assistenzärzte in Weiterbildung zu ihren Praktika im Medizinstudium befragt. Der standardisierte und selektiv programmierte Fragebogen umfasste bis zu 160 Fragen, die auf einer 5‑stufigen Likert-Skala mit 1 – „ich stimme gar nicht zu“ bis 5 – „ich stimme voll und ganz zu“ zu beantworten waren. Hinzu kamen noch Fragen mit Mehrfachnennungen. Damit ist die Befragung zahlenmäßig die zweitgrößte, jedoch zeitgleich die umfangreichste Evaluation der Praktika deutscher Medizinstudierender [16]. Zusätzlich gaben die Teilnehmenden soziodemografische Daten, also Alter, Geschlecht, Angaben zum Studienabschnitt (Anzahl der Semester), Studienort und Studiengang (Regel- oder Modellstudiengang) an.

Während die Gesamtumfrage 9079 Datensätze enthielt, befanden sich 4146 Teilnehmende im 1. bis 3. klinischen Jahr und hatten bereits eine Famulatur absolviert. Die in dieser Arbeit weiter betrachtete Subgruppe von 479 Studierenden (19 % der Evaluationen einer Famulatur) machte Angaben zu ihrer mindestens 4‑wöchigen Famulatur im Fachgebiet der Anästhesiologie. Hierbei wurden die Angaben zu Items in den 6 inhaltlichen Kategorien analysiert: 1. Integration in das Team, 2. Erwerb von Kompetenzen, 3. Lehrende, 4. Qualität und 5. Struktur der Lehre sowie 6. die Zufriedenheit mit der Famulatur.

**Figure 1:**
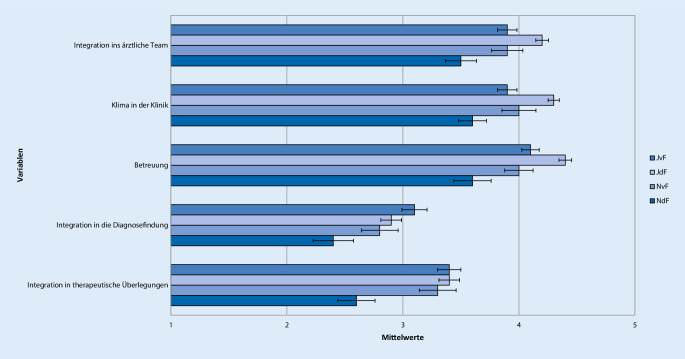

**Figure 2:**
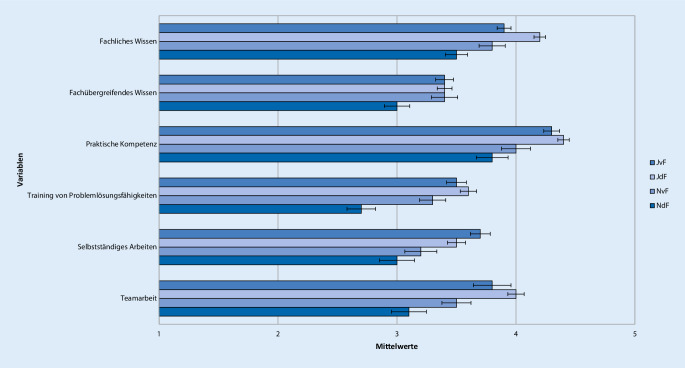

**Figure 3:**
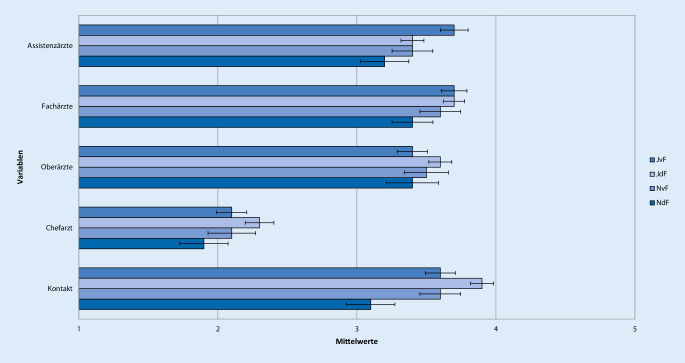

**Figure 4:**
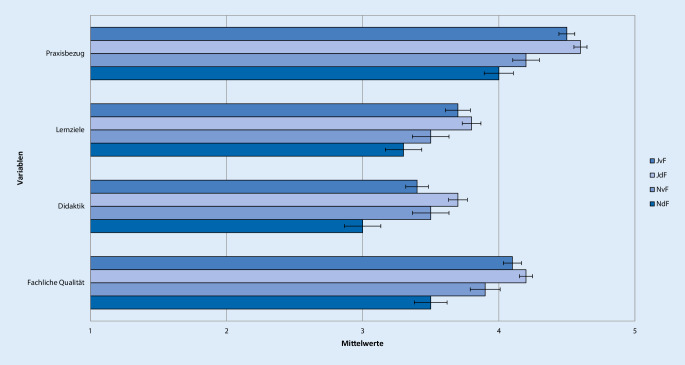

Antworten auf der 5‑stufigen Likert-Skala mit 1 oder 2 wurden als „ich stimme nicht zu“, mit 3 als „neutral“ und mit 4 oder 5 als „ich stimme zu“ zusammengefasst. Um Unterschiede in der Bewertung der Famulatur und somit Kriterien zur Entscheidung der Studierenden für oder gegen ein weiteres Interesse an Anästhesiologie zu evaluieren, wurden 4 Gruppen gebildet. Neben der Entscheidung für oder gegen ein Wahltertial Anästhesiologie im PJ wurde auch der Zeitpunkt der Entscheidungsfindung berücksichtigt. Die Unterteilung erfolgte anhand der Beantwortung der Frage „Können Sie sich vorstellen, das Fachgebiet Ihrer Famulatur auch für das Wahltertial im PJ zu wählen?“ in folgende 4 Gruppen: „Ja, diese Entscheidung habe ich schon vor der Famulatur getroffen“ (JvF, *n* = 144), „Ja, diese Entscheidung habe ich jetzt durch die Famulatur getroffen“ (JdF, *n* = 212), „Nein, diese Entscheidung habe ich schon vor der Famulatur getroffen“ (NvF, *n* = 67) oder „Nein, diese Entscheidung habe ich jetzt durch die Famulatur getroffen“ (NdF, *n* = 56; Tab. 1, 2, 3, 4, 5 und 6; Abb. 1, 2, 3, 4, 5 und 6).

**Table 1:** 

Aussagen/Fragen/Items	Analyse				Gesamtantworten 1 und 2 [%]	Gesamtantworten 3 [%]	Gesamtantworten 4 und 5 [%]
	ANOVA		Paarweise Vergleiche				
	F	*p*	JvF vs. JdF	JdF vs. NdF			
Integration ins ärztliche Team	7,25	<0,001	0,223	<0,001	8,8	17,3	73,9
Klima in der Klinik	10,98	<0,001	<0,001	<0,001	7,2	14,5	78,3
Betreuung	11,81	<0,001	0,006	<0,001	5,9	14,1	80
Integration in die Diagnosefindung	4,43	0,004	0,079	0,042	38,7	26,8	34,5
Integration in therapeutische Überlegungen	5,26	0,001	1,000	0,001	27,8	21,1	51,2

P- und F‑Wert der analysierten Gruppen: *JvF *Entscheidung zum PJ-Wahltertial Anästhesie vor der Famulatur; *JdF* Entscheidung zum PJ-Wahltertial Anästhesie durch die Famulatur; *NvF* Entscheidung gegen PJ-Wahltertial Anästhesie vor der Famulatur; *NdF* Entscheidung gegen PJ-Wahltertial Anästhesie durch die Famulatur. Paarweise Vergleiche der Subgruppen JvF vs. JdF sowie JdF vs. NdF. Die Gesamtantworten wurden in der Likert-Skala zusammengefasst: 1 und 2 „unzufrieden“, 3 „unentschieden“, 4 und 5 „zufrieden“

**Table 2:** 

Aussagen/Fragen/Items	Analyse				Gesamtantworten 1 und 2 [%]	Gesamtantworten 3 [%]	Gesamtantworten 4 und 5 [%]
	ANOVA		Paarweise Vergleiche				
	F	*p*	JvF vs. JdF	JdF vs. NdF			
Fachliches Wissen	13,49	<0,001	0,050	<0,001	3,3	19,7	77
Fachübergreifendes Wissen	4,16	0,006	1,000	0,005	15,8	36,5	47,7
Praktische Kompetenz	12,24	<0,001	1,000	<0,001	4,7	11,3	84
Training von Problemlösungsfähigkeiten	10,45	<0,001	1,000	<0,001	19,4	29,3	51,3
Selbstständiges Arbeiten	5,64	0,001	1,000	0,023	22	27,5	50,5
Teamarbeit	11,4	<0,001	1,000	<0,001	9,9	28,1	62

P- und F‑Wert der analysierten Gruppen: *JvF *Entscheidung zum PJ-Wahltertial Anästhesie vor der Famulatur; *JdF* Entscheidung zum PJ-Wahltertial Anästhesie durch die Famulatur; *NvF* Entscheidung gegen PJ-Wahltertial Anästhesie vor der Famulatur; *NdF* Entscheidung gegen PJ-Wahltertial Anästhesie durch die Famulatur. Paarweise Vergleiche der Subgruppen JvF vs. JdF sowie JdF vs. NdF. Die Gesamtantworten wurden in der Likert-Skala zusammengefasst: 1 und 2 „unzufrieden“, 3 „unentschieden“, 4 und 5 „zufrieden“

**Table 3:** 

Aussagen/Fragen/Items	Analyse				Gesamtantworten 1 und 2 [%]	Gesamtantworten 3 [%]	Gesamtantworten 4 und 5 [%]
	ANOVA		Paarweise Vergleiche				
	F	*p*	JvF vs. JdF	JdF vs. NdF			
Assistenzärzte	2,16	0,092	0,328	1,000	22,2	24,8	53
Fachärzte	1,6	0,190	1,000	0,210	14,5	23,6	61,9
Oberärzte	1,12	0,340	0,657	1,000	24,1	23,1	52,7
Chefarzt	2,05	0,106	0,508	0,192	67,1	11,2	21,6
Kontakt	5,27	0,001	0,397	0,001	17,9	19,8	62,3

P- und F‑Wert der analysierten Gruppen: *JvF *Entscheidung zum PJ-Wahltertial Anästhesie vor der Famulatur; *JdF* Entscheidung zum PJ-Wahltertial Anästhesie durch die Famulatur; *NvF* Entscheidung gegen PJ-Wahltertial Anästhesie vor der Famulatur; *NdF* Entscheidung gegen PJ-Wahltertial Anästhesie durch die Famulatur. Paarweise Vergleiche der Subgruppen JvF vs. JdF sowie JdF vs. NdF. Die Gesamtantworten wurden in der Likert-Skala zusammengefasst: 1 und 2 „unzufrieden“, 3 „unentschieden“, 4 und 5 „zufrieden“

**Table 4:** 

Aussagen/Fragen /Items	Analyse				Gesamtantworten 1 und 2 [%]	Gesamtantworten 3 [%]	Gesamtantworten 4 und 5 [%]
	ANOVA		Paarweise Vergleiche				
	F	*p*	JvF vs. JdF	JdF vs. NdF			
Praxisbezug	12,36	<0,001	1,000	<0,001	0,8	10,9	88,3
Lernziele	5,19	0,002	1,000	0,003	12,8	26,6	60,6
Didaktik	9,16	<0,001	0,040	<0,001	14,4	34,3	51,3
Fachliche Qualität	10,14	<0,001	1,000	<0,001	2,5	20,6	76,9

P- und F‑Wert der analysierten Gruppen: *JvF *Entscheidung zum PJ-Wahltertial Anästhesie vor der Famulatur; *JdF* Entscheidung zum PJ-Wahltertial Anästhesie durch die Famulatur; *NvF* Entscheidung gegen PJ-Wahltertial Anästhesie vor der Famulatur; *NdF* Entscheidung gegen PJ-Wahltertial Anästhesie durch die Famulatur. Paarweise Vergleiche der Subgruppen JvF vs. JdF sowie JdF vs. NdF. Die Gesamtantworten wurden in der Likert-Skala zusammengefasst: 1 und 2 „unzufrieden“, 3 „unentschieden“, 4 und 5 „zufrieden“

**Table 5:** 

Aussagen/Fragen/Items	Analyse				Gesamtantworten 1 und 2 [%]	Gesamtantworten 3 [%]	Gesamtantworten 4 und 5 [%]
	ANOVA		Paarweise Vergleiche				
	F	*p*	JvF vs. JdF	JdF vs. NdF			
Aufbau und Struktur	7,42	<0,001	0,021	<0,001	30,2	22,6	47,3
Mentorenprogramm	4,71	0,003	1,000	0,001	23,2	44,2	32,7
Manuelle Fertigkeiten	7,06	<0,001	1,000	<0,001	4,9	7	88,1
Patientenferne Tätigkeit	4,3	0,005	0,903	0,005	88,2	7,6	4,2
„Bedside teaching“	4,5	0,004	1,000	0,009	37,2	19,2	43,5

P- und F‑Wert der analysierten Gruppen: *JvF *Entscheidung zum PJ-Wahltertial Anästhesie vor der Famulatur; *JdF* Entscheidung zum PJ-Wahltertial Anästhesie durch die Famulatur; *NvF* Entscheidung gegen PJ-Wahltertial Anästhesie vor der Famulatur; *NdF* Entscheidung gegen PJ-Wahltertial Anästhesie durch die Famulatur. Paarweise Vergleiche der Subgruppen JvF vs. JdF sowie JdF vs. NdF. Die Gesamtantworten wurden in der Likert-Skala zusammengefasst: 1 und 2 „unzufrieden“, 3 „unentschieden“, 4 und 5 „zufrieden“

**Table 6:** 

Aussagen/Fragen/Items	Analyse				Gesamtantworten 1 und 2 [%]	Gesamtantworten 3 [%]	Gesamtantworten 4 und 5 [%]
	ANOVA		Paarweise Vergleiche				
	F	*P*	JvF vs. JdF	JdF vs. NdF			
Wertung des Gesamteindrucks	22,97	<0,001	0,001	<0,001	3,7	10,4	85,9
Zufriedenheit mit Famulatur	15,13	<0,001	0,002	<0,001	8,2	11,3	80,5
Zufriedenheit mit Entscheidung zum Arzt	0,14	0,937	1,000	1,000	3,3	10,2	86,5
Weiterempfehlung des Medizinstudiums an Freunde	0,7	0,552	1,000	1,000	16,2	24,8	59,1

P- und F‑Wert der analysierten Gruppen: *JvF *Entscheidung zum PJ-Wahltertial Anästhesie vor der Famulatur; *JdF* Entscheidung zum PJ-Wahltertial Anästhesie durch die Famulatur; *NvF* Entscheidung gegen PJ-Wahltertial Anästhesie vor der Famulatur; *NdF* Entscheidung gegen PJ-Wahltertial Anästhesie durch die Famulatur. Paarweise Vergleiche der Subgruppen JvF vs. JdF sowie JdF vs. NdF. Die Gesamtantworten wurden in der Likert-Skala zusammengefasst: 1 und 2 „unzufrieden“, 3 „unentschieden“, 4 und 5 „zufrieden“

**Figure 5:**
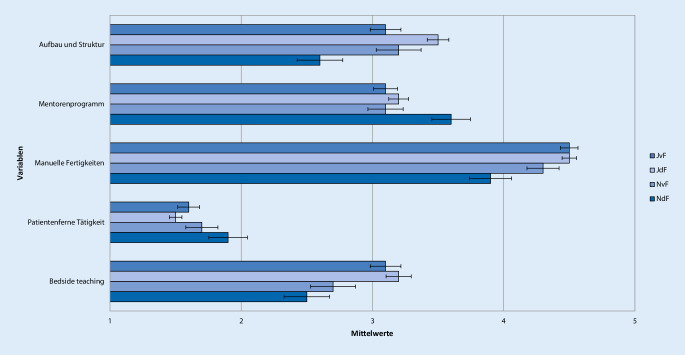

**Figure 6:**
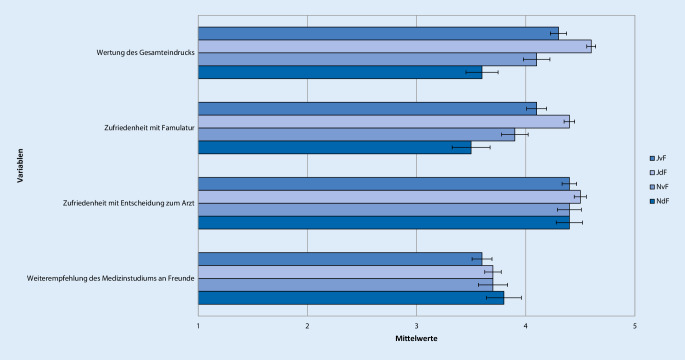

Die statistische Auswertung erfolgte mittels SPSS 22.0 (IBM Corp., Armonk, USA). Dazu wurden eine multivariate Varianzanalyse sowie univariate ANOVA mit dem Zwischensubjektfaktor Gruppe („JvF“ gegenüber „NvF“ gegenüber „JdF“ gegenüber „NdF“) für die einzelnen Evaluationsitems durchgeführt. Außerdem wurden Bonferroni-korrigierte Post-hoc-Vergleiche durchgeführt.

## Ergebnisse

Das Durchschnittsalter der Teilnehmenden lag bei 25,8 Jahren (SD = 3,32; Range: 20–52); etwa die Hälfte (51 %) davon war weiblich. Alle medizinischen Fakultäten und Studienorte wurden erreicht. Insgesamt entschieden sich 56 % der Studienteilnehmenden aktiv durch die in der Famulatur gemachten Erfahrungen für oder gegen ein PJ-Tertial im gleichen Fach. Das anästhesiologische Praktikum konnte dabei 79 % der zuvor unentschiedenen Studierenden (*n* = 268) für das Fachgebiet der Anästhesiologie gewinnen. In den 4 Gruppen fanden sich insbesondere bedeutsame Unterschiede im Bereich der „Integration ins Team“, des „Erwerbs von Kompetenzen“ sowie der „Qualität der Lehre“. Ein geringerer Anteil von 14 % aller Studienteilnehmenden war bereits vor der Famulatur ablehnend gegenüber weiteren Praktika in der Anästhesiologie eingestellt.

### Integration in das Team

Die überwiegende Mehrheit (74 %) aller Famuli vergab im Bereich der Integration in das ärztliche Team (sehr) gute Bewertungen. Vier von 5 Studierenden erlebten ein (sehr) gutes Klima in der anästhesiologischen Klinik, ebenso viele fühlten sich (sehr) gut durch die Ärzte betreut. Während über ein Drittel der Famuli nicht in die Diagnosefindung einbezogen wurden, äußerten sich weitere 27 % neutral zu dieser Frage. Die Integration in therapeutische Überlegungen erfolgte bei etwa der Hälfte der Famuli gut oder sehr gut.

Die Gruppenvergleiche ergaben einen signifikanten Einfluss aller befragten Items auf die Entscheidung, aufgrund der Famulatur auch ein PJ-Tertial in der Anästhesiologie zu absolvieren (alle *p* ≤ 0,004). Sowohl die Integration in das ärztliche Team (*p* < 0,001), das Klima in der Klinik (*p* < 0,001), die Betreuung (*p* < 0,001) als auch die Integration in therapeutische Überlegungen (*p* = 0,001) bewertete die Gruppe JdF im Einzelvergleich hochsignifikant besser als die Gruppe NdF. Des Weiteren bewertete die Gruppe JdF das Klima in der Klinik (*p* < 0,001) und die Betreuung in der Famulatur (*p* = 0,006) signifikant besser als die Gruppe JvF (Abb. 1; Tab. 1).

### Erwerb von Kompetenzen

Mehr als drei Viertel der Studierenden gaben an, fachliches Wissen (77 %) und praktische Kompetenzen/Praxiserfahrung (84 %) in (sehr) hohem Maße erworben zu haben. Demgegenüber erwarb jede*r zweite Teilnehmende fachübergreifendes Wissen sowie problemlösende und analytische Fähigkeiten und gab an, während der Tätigkeiten selbstständig gearbeitet zu haben. Etwa zwei Drittel (62 %) der Famuli kooperierten gut mit dem ärztlichen Team.

Das Ausmaß des Kompetenzerwerbes unterschied sich zwischen den Studierenden, die sich für oder gegen das PJ in der Anästhesiologie aussprachen. So zeigte sich in den Gruppenvergleichen, dass besonders die Faktoren fachliches Wissen (*p* < 0,001), Praxiserfahrung (*p* < 0,001), die Vermittlung von Teamarbeitsfähigkeit (*p* < 0,001) als auch der Erwerb fachlicher problemlösender und analytischer Fähigkeiten (*p* < 0,001) von den einzelnen Gruppen signifikant unterschiedlich wahrgenommen wurden. Dabei bewertete insbesondere die Gruppe JdF alle Items signifikant besser als die Gruppe NdF (*p* ≤ 0,023) (Abb. 2; Tab. 2).

### Lehrende

Den größten Wissenszuwachs erhielten die anästhesiologischen Famuli durch die Gruppe der Fachärzte. Zusätzlich gab etwa die Hälfte der Famuli an, auch von den Oberärzten und Assistenzärzten zu lernen, während nur etwa jede und jeder Fünfte die Chefärzte als Hauptlehrende empfand. Insgesamt bewerteten etwa zwei Drittel der Famuli (62 %) den Kontakt zu den Lehrenden mit „gut“ oder „sehr gut“. Signifikante Unterschiede wiesen die Gruppen, welche sich durch die Famulatur für oder gegen Anästhesiologie entschieden hatten, bezüglich des engen Kontakts zu den Lehrenden auf (*p* = 0,001), mit dem sich jedoch insgesamt 38 % aller Befragten nicht zufrieden zeigten (Abb. 3; Tab. 3).

### Qualität der Lehre

Besonders positiv wurden Praxisbezug und die fachliche Qualität der Lehre eingeschätzt. Die didaktische Qualität der Lehre beurteilten nur etwas mehr als die Hälfte (51 %) der Famuli mit (sehr) gut. Die Lernziele wiederum wurden von 61 % der Studierenden erreicht. Insgesamt bewertete die Gruppe JdF die Qualität der Lehre signifikant besser als die Gruppe NdF (alle Items *p* < 0,003) (Abb. 4; Tab. 4).

### Struktur der Lehre

Der Großteil der Famuli war sich einig, manuelle Fertigkeiten an den Patienten erlernt zu haben. Nur 4 % der Famuli beschrieben ihre Tätigkeiten als „zu patientenfern“. Knapp die Hälfte der Famuli stufte den Aufbau und die Struktur der Famulatur als (sehr) gut ein. Nur ein Drittel war mit dem Mentorenprogramm zufrieden, während sich 44 % der Famuli mit dem „bedside teaching“ zufrieden zeigten.

Insgesamt bewertete die JdF-Gruppe alle Items signifikant besser als die Gruppe NdF (*p* ≤ 0,005). Diejenigen, die durch die Famulatur für das Fachgebiet gewonnen werden konnten, bewerteten den Aufbau und die Struktur ihrer Famulatur signifikant besser als diejenigen, die schon vor der Famulatur ein PJ-Tertial in der Anästhesiologie absolvieren wollten (JvF vs. JdF *p* = 0,021) (Abb. 5; Tab. 5).

### Zufriedenheit

Sehr zufrieden oder zufrieden mit ihrer Famulatur zeigten sich 81 % der Studierenden, 86 % hatten einen positiven Gesamteindruck. Die meisten der Befragten (87 %) gaben nach der Famulatur an, zufrieden mit der Entscheidung für den Arztberuf zu sein. Weniger als zwei Drittel der Famuli waren bereit, den ärztlichen Beruf Freunden und Bekannten weiterzuempfehlen (59 %).

Bezüglich des Gesamteindrucks und der Zufriedenheit mit der Famulatur, ergaben sich im paarweisen Vergleich signifikant bessere Bewertungen durch die Gruppe JdF im jeweiligen Vergleich zu den Gruppen JvF als auch NdF (*p* ≤ 0,002) im bewerteten Praktikum (Abb. 6; Tab. 6).

## Diskussion

Diese Studie überprüfte, inwiefern frühe klinische Erfahrungen im Medizinstudium in Form von curricularen Praktika die Entscheidung der Studierenden beeinflussten, auch das PJ-Wahltertial im gleichen Fachgebiet zu belegen. Weiterhin wurden Aspekte herausgearbeitet, die für die anästhesiologische Famulatur besonders relevant sind. Aus diesen Ergebnissen lässt sich auf einen möglichen Zusammenhang zwischen einer zufriedenstellenden Famulatur und dem weiteren Interesse am Fachgebiet Anästhesiologie schließen. Folglich ist die Famulatur eine interessante Möglichkeit, um Studierende für die Anästhesiologie zu gewinnen.

Besonders bedeutend in der studentischen Wertung zeigte sich die gute Integration der Famuli in das ärztliche Team. Auch das Klima in der anästhesiologischen Klinik und die ärztliche Betreuung beurteilten die 4 Gruppen der Studierenden unterschiedlich, wodurch sich diese als relevante Faktoren für oder gegen ein weiteres Interesse an Anästhesiologie werten lassen. Im Vergleich zu anderen Fachgebieten zeigt sich, dass Famuli in Orthopädie, Radiologie oder Chirurgie das Klima in der Klinik und die ärztliche Betreuung ebenfalls positiv werten [5, 19, 20]. Daraus schlussfolgern die Autorinnen und Autoren, dass die persönliche Betreuung in der Famulatur nicht zu kurz kommen sollte. Im Gegensatz dazu bewerteten die Teilnehmenden therapeutische Überlegungen und ihre Teilnahme an der Diagnosefindung als weniger interessant, da diese von allen 4 Gruppen, auch der Gruppe „JdF“, deutlich schlechter bewertet wurden.

Als motivierend bewerteten die Teilnehmenden klinische Erfolgserlebnisse, bei denen sie ihre praktischen Fähigkeiten verbessern konnten. Diese scheinen also für die Fachgebietswahl ebenfalls relevant. Genauso bedeutend wie die Vermittlung spezifischen Fachwissens war der Umstand, dass Studierende, die aufgrund ihrer Famulatur das Fach Anästhesiologie als PJ-Wahl-Tertial wählen möchten, einen engeren Kontakt zu den Lehrenden erlebten. Wie auch in Studienergebnissen anderer Fachgebiete hatte der Weiterbildungsgrad der Lehrenden vermutlich wenig Einfluss auf die Begeisterung der Studierenden für die Anästhesiologie und wurde von allen Gruppen ähnlich bewertet [27]. Die Relevanz guter Lehrender, die als positive Rollenmodelle das Interesse am Fachgebiet fördern, zeigen aktuelle Publikationen und heben auch z. B. die interdisziplinäre Zusammenarbeit positiv heraus [27, 29].

Verglichen mit anderen Variablen wurde die Didaktik von allen Gruppen schlechter bewertet. Hieraus ergeben sich konkrete Ansatzstellen, durch kompetenzbasierte Konzepte didaktischer Trainings die Lehrkompetenz der betreuenden Kollegen zu verbessern. Zur Veranstaltung dieser Trainings gibt es bereits fortschrittliche Konzepte, in welchen innerhalb von Kleingruppen kompetenzbasierte Unterrichtseinheiten und deren anschließende Reflexion durchgeführt werden. Diese Erfahrungen sollen dazu dienen, eigene Unterrichtskonzepte zu erstellen und die bisherigen Ansätze qualitativ zu verbessern [8]. Da vorwiegend Assistenz- und Fachärzte Wissen an die Studierenden vermitteln, dürften besonders diese beiden Berufsgruppen von Schulungen profitieren. Insgesamt fand im Vergleich, möglicherweise fachgebietsbedingt, wenig „bedside teaching“ in den untersuchten Famulaturen statt [15, 29]. Eine Erklärung für den geringen Anteil an „bedside teaching“ könnte sein, dass viele Anästhesisten hauptsächlich im OP und weniger auf den Stationen tätig sind. Hier ist zu vermuten, dass die Studierenden die Lehre im OP nicht als „bedside teaching“ klassifizierten, obwohl Lehre im OP am Patienten vom Begriff des „bedside teaching“ umfasst wird. Es ist nicht auszuschließen, dass die Fragestellung in diesem Zusammenhang fehlleitend war.

Diejenigen, die sich nach der Famulatur ein PJ-Wahltertial in der Anästhesiologie vorstellen konnten, waren auch deutlich zufriedener mit Aufbau und Struktur der Famulatur und durften mehr manuelle Tätigkeiten ausüben als diejenigen, die aufgrund der Famulatur ihr weiteres praktisches Interesse an der Anästhesiologie verloren. Dies deutet auf wachsende Anforderungen an den zukünftigen Arbeitsplatz und einen strukturierten Arbeitsalltag hin. Auch eine Analyse [13] des Ärztemangels in der Anästhesiologie und ein Review [26], das sich mit intergenerationalen Differenzen in der Anästhesiologie befasst, kommen zu dem Schluss, dass die neue Generation angehender Ärztinnen und Ärzte für den klinischen Alltag zukünftig anders zu motivieren sein wird. Um die Struktur in der Famulatur zu verbessern, bietet sich die Erstellung einer „Famulatur-Checkliste“ mit entsprechenden Lernzielen an, wobei man sich an Inhalten aus Fachliteratur zur Lehre orientieren kann [11].

Im Vergleich mit anderen Fachgebieten lässt sich hinsichtlich der Erwartungen an den späteren Arbeitsalltag der Studierenden z. T bereits sehr früh eine unterschiedliche Interessenlage zu den unterschiedlichen Fachgebieten ablesen. So zeigt sich das anästhesiologische Fachgebiet im Unterschied zu den chirurgischen Alternativen bei Studierenden beliebter, welche höhere Präferenzen im Bereich der Teilzeitarbeit bzw. Familienfreundlichkeit aufweisen [5, 16, 19, 20]. Insbesondere durch die steigende Anzahl von Medizinstudentinnen muss sich das Fachgebiet für Anästhesiologie an die wachsende Forderung nach Teilzeitarbeitsmöglichkeiten und Kinderbetreuung anpassen. Die vermehrte Teilzeitarbeit in der Anästhesiologie erklärt den bestehenden Ärztemangel trotz steigender Ärztezahl [25]. Nationale und internationale Studienergebnisse zeigen Lifestyleveränderungen im zeitlichen Verlauf und werten die Ansprüche an eine persönliche Arbeitsplatzgestaltung als in ihrer Wichtigkeit ansteigend [4, 9, 13, 26].

Im Gegensatz zu anderen Fachgebieten wie Radiologie, Orthopädie/Unfallchirurgie und Chirurgie liegen bisher wenig Informationen zu curricularen Praktika an deutschen Universitäten im Fachgebiet Anästhesiologie vor. Bekannt ist, dass Studierende, die ihre Praktika in anderen Fachgebieten absolvierten, ebenso hohe Erwartungen an deren Gestaltung zeigten [5, 19, 20]. Gründe gegen weitere Praktika waren auch in den anderen Fachgebieten Mängel in Qualität der Lehre und dem „bedside teaching“, während sich Studierende, die eine gute Integration ins Team erlebten und praktische Fähigkeiten erwarben, zufriedener mit dem Praktikum zeigten [15].

Da es sich bei dieser Online-Befragung um eine Querschnittsstudie handelt, lassen die auf einer 5‑stufigen Likert-Skala bewerteten Daten limitierend keine Rückschlüsse auf kausale Zusammenhänge zwischen der Zufriedenheit der Befragten und deren tatsächlichen späteren Wahl des PJ-Tertials zu. Dennoch geben die Ergebnisse Hinweise auf Bereiche, deren Verbesserung zu einer Zufriedenheitssteigerung der Famuli führen können. Um genauere Gründe der Befragten für oder gegen die Wahl eines PJ-Tertials zu erforschen, wäre eine kleinere Stichprobenbefragung mit einer zusätzlichen Freitexteingabe möglich.

Weiterhin ist zu erwähnen, dass es sich bei den erhobenen Daten um eine selbstselektive Stichprobe handelt, die eine fehlende Generalisierbarkeit der Ergebnisse vermuten lässt [6]. Da sich jedoch die soziodemografischen Daten mit denen anderer umfangreicher Befragungen der Medizinstudierenden in Deutschland und der Grundgesamtheit der Studierenden decken, ist davon auszugehen, dass die Ergebnisse die typischen Ansichten anästhesiologischer Famuli wiedergeben [6, 10, 24].

Des Weiteren ist zu bedenken, dass Studierende ihre Famulaturen auch ohne klinische Vorkenntnisse absolvieren dürfen, sobald sie das Physikum bestanden haben. Die vorliegende Umfrage erhob alle sich im klinischen Abschnitt befindlichen Famuli, die angaben, eine mindestens 4‑wöchige Famulatur gemacht zu haben, ohne weitere Binnendifferenzierung. Dabei kommt möglicherweise die Unterscheidung zwischen Studierenden zu kurz, welche vor dem Praktikum die theoretischen Inhalte des Faches Anästhesiologie bereits erfolgreich absolviert hatten, und jenen, die noch keine Kenntnisse in diesem Fachgebiet besaßen.

Weiterführend wäre es auch von Interesse zu erfahren, wie die Studierenden das Wahltertial Anästhesiologie in ihrem PJ wahrnehmen.

Aufgrund des bestehenden Ärztemangels, der sowohl die Anästhesiologie als auch andere Fachgebiete betrifft, empfehlen die Autorinnen und Autoren, mehr Wert auf eine gute Gestaltung und Evaluation der curricularen Praktika von Medizinstudierenden zu legen. Deshalb wäre eine Folgestudie sinnvoll, in der untersucht wird, inwiefern durch eine gute Lehre, beispielsweise gefördert durch didaktische Weiterbildung, und angemessene Struktur der Famulatur, beispielsweise anhand von Checklisten und einem ausgearbeiteten Curriculum, mehr Studierende für das Fachgebiet Anästhesiologie gewonnen werden können. Hierbei ließe sich auch überprüfen, inwiefern innovative Lehrmethoden während der Famulatur zur Nachwuchsgenerierung beitragen. Mathis et al. entwickelten eine Kurzanleitung für Studierende mit webbasierter Videoanleitung, um erste praktische Fähigkeiten auszubilden und die Vermittlung von Fachwissen in der Anästhesiologie zu fördern [23]. Diese unterstützenden Lehrmethoden sind empfehlenswert, um das Interesse am Fach zu erhöhen. Jedoch ist dabei auf eine Ausgewogenheit zwischen Simulation und klinischen Kursen zu achten, da Studierende, die ihr Wissen ausschließlich über E‑Learning-Simulationen vermittelt bekommen, schlechter abschneiden als jene, die ihre Fähigkeiten im Klinikalltag lernen und anwenden können [22]. Zusätzlich könnte durch „Train-the-trainer“-Weiterbildungen die Qualität der Lehre im Praktikum verbessert werden [7, 20]. Dabei könnten die von den Befragten als weniger gut eingestuften Kriterien, wie z. B. die Didaktik und die Vermittlung fachlicher problemlösender und analytischer Fähigkeiten, als Grundlage für ein Weiterbildungskonzept dienen.

## Schlussfolgerung

Durch eine zufriedenstellende Famulatur innerhalb der Anästhesiologie können Studierende bereits zu einem frühen Zeitpunkt des Studiums für die Anästhesiologie begeistert werden. Wer auch das PJ in der Anästhesiologie machen wollte, bewertete die Famulatur besser, was möglicherweise auf eine bessere Ausbildung im Rahmen der Famulatur zurückzuführen ist. Besonders positiv bewerteten die Teilnehmenden der Studie eine starke Integration in das Team sowie den Erwerb praktischer Kompetenzen und von Fachwissen. Demgegenüber beurteilten die Studierenden Didaktik und Praxisbezug in der Lehre als verbesserungswürdig. Um diese Mängel zu beseitigen, sind Investitionen in die Lehre und die klare Formulierung von Lernzielen unabdingbar. Dadurch könnten Studierende stärker dazu bewegt werden, sich für die Anästhesiologie als Fachgebiet zu entscheiden, was wiederum dem Nachwuchsmangel entgegenwirkt. Um angehende Ärztinnen und Ärzte für die Anästhesiologie zu gewinnen, sollte das ärztliche Team die Famulatur möglichst lehrreich und praxisnah gestalten.

## Fazit für die Praxis

- Die anästhesiologische Famulatur und die spätere Spezialisierungspräferenz hängen zusammen.
- Qualität und Struktur der Lehre wirken sich auf die Zufriedenheit der Studierenden aus.
- Die Vermittlung praktischer Kompetenzen und von Fachwissen in der Famulatur kann die Nachwuchsgewinnung fördern.
- Mit dem anästhesiologischen Praktikum zufriedene Famuli waren gut in das ärztliche Team integriert und sowohl in Diagnosefindungen als auch Therapieplanung einbezogen.
- Um die Lehre in der Famulatur und die didaktische Kompetenz der Lehrenden zu verbessern, werden didaktische Fort- und Weiterbildungen vorrangig für die lehrenden Assistenz- und Fachärzte empfohlen. Eine verbesserte Strukturierung der Famulatur kann durch eine „Famulatur-Checkliste“ mit entsprechenden Lernzielen erreicht werden.
